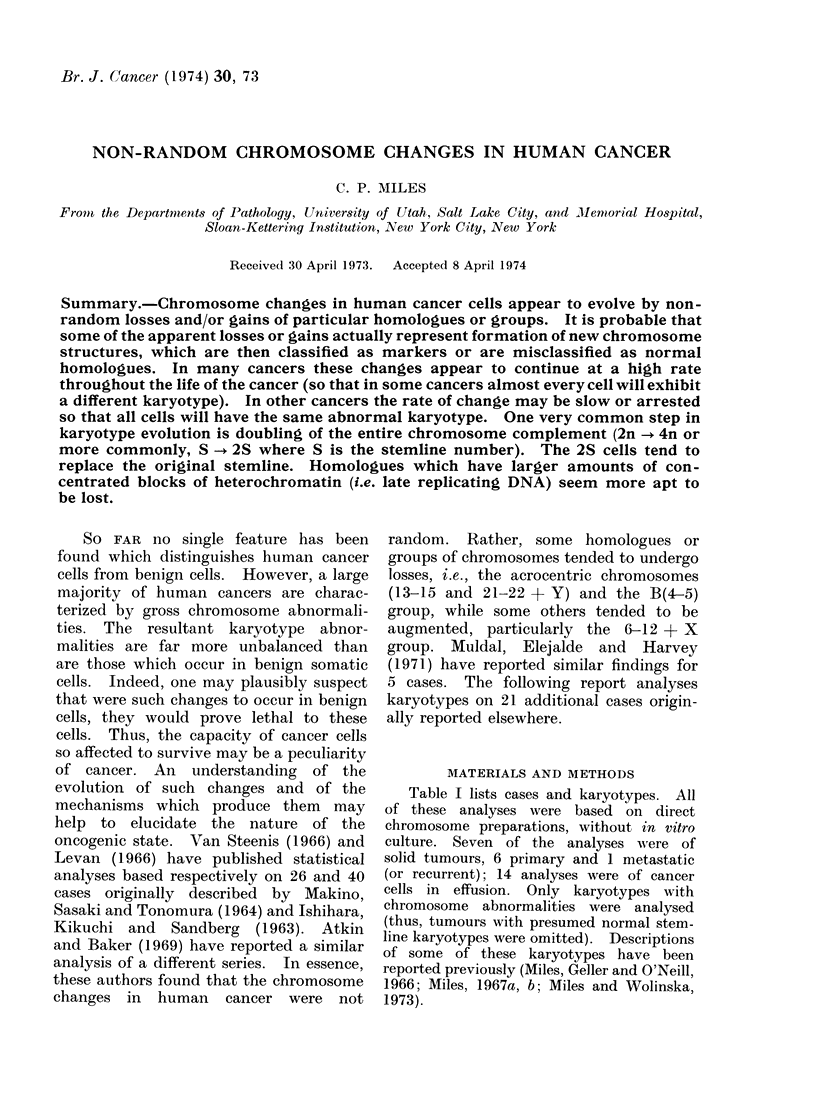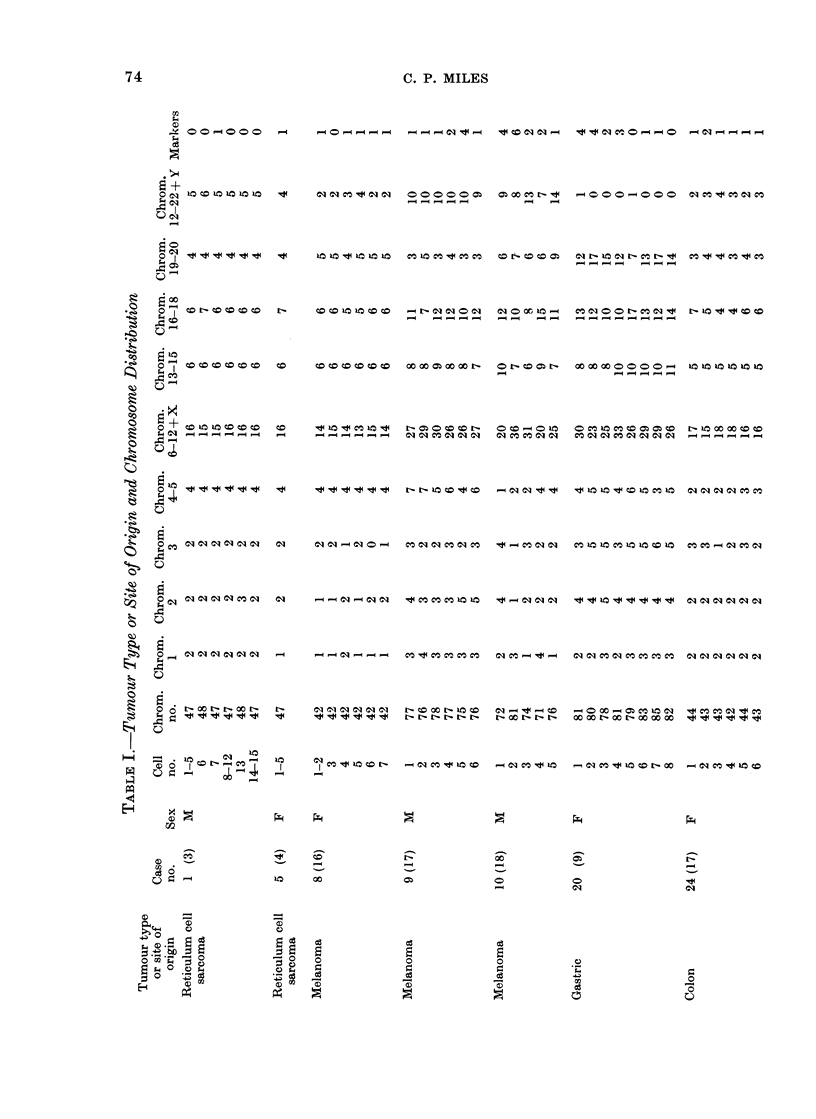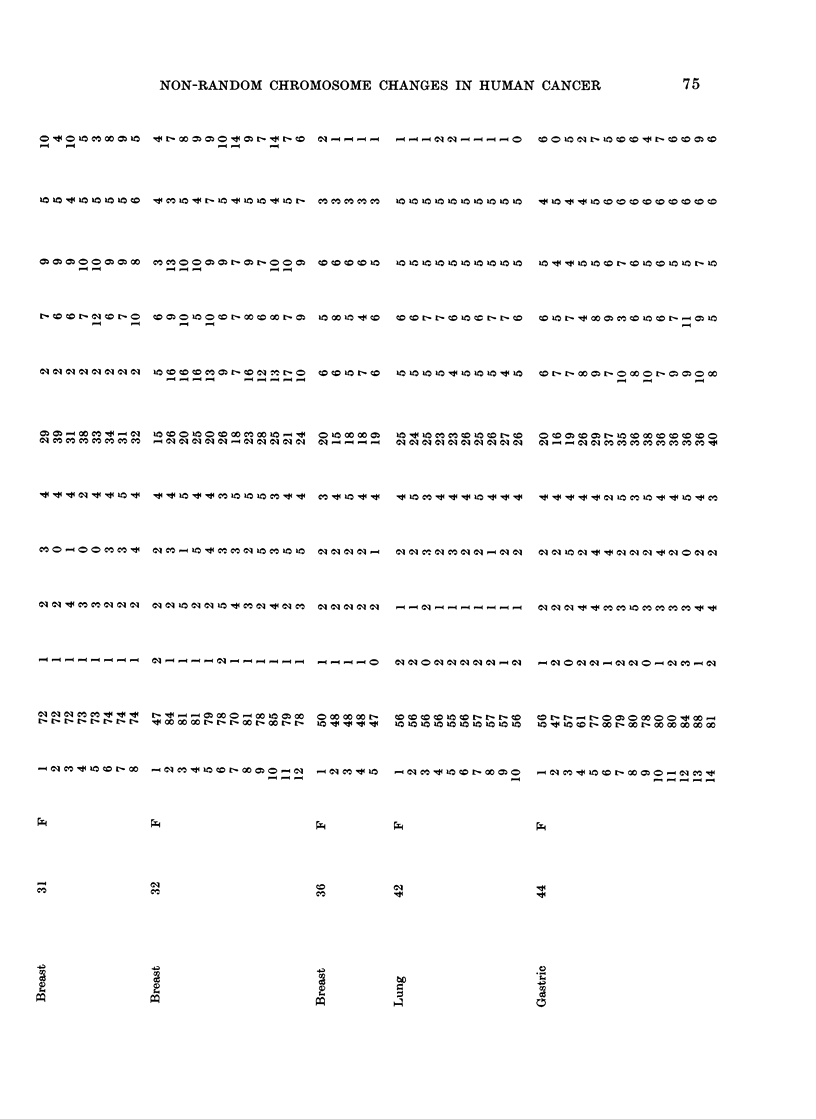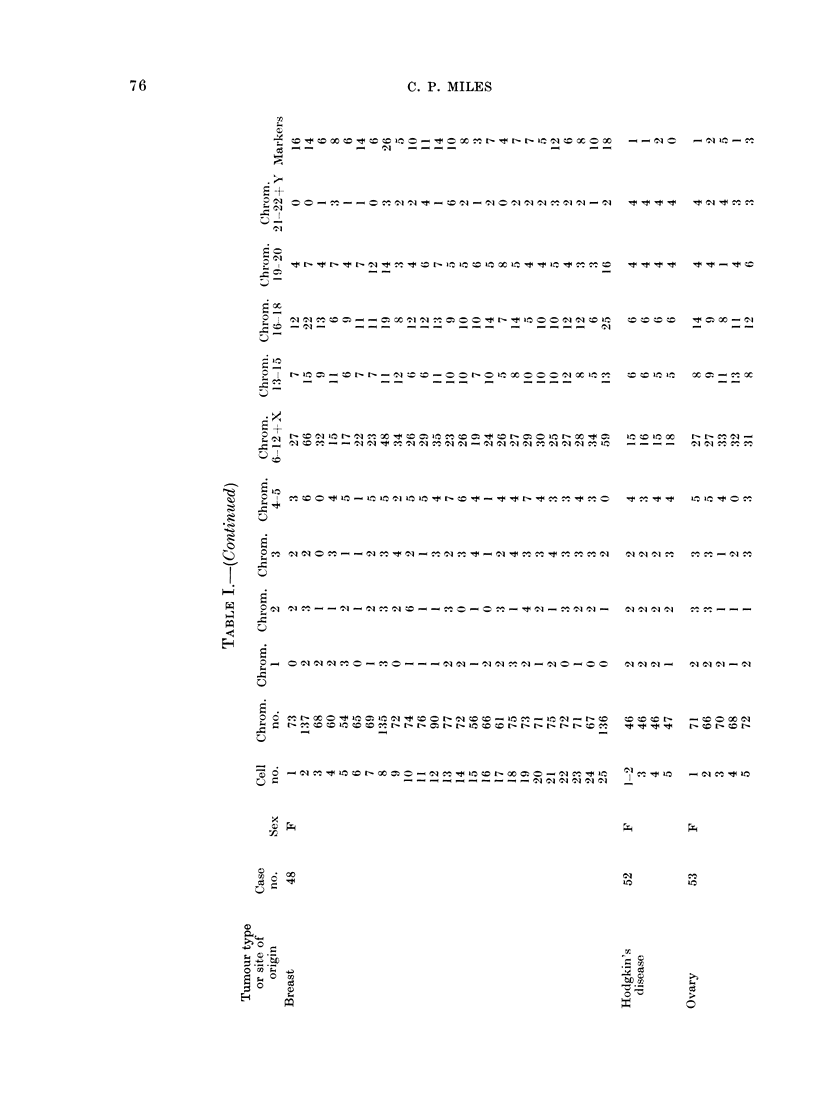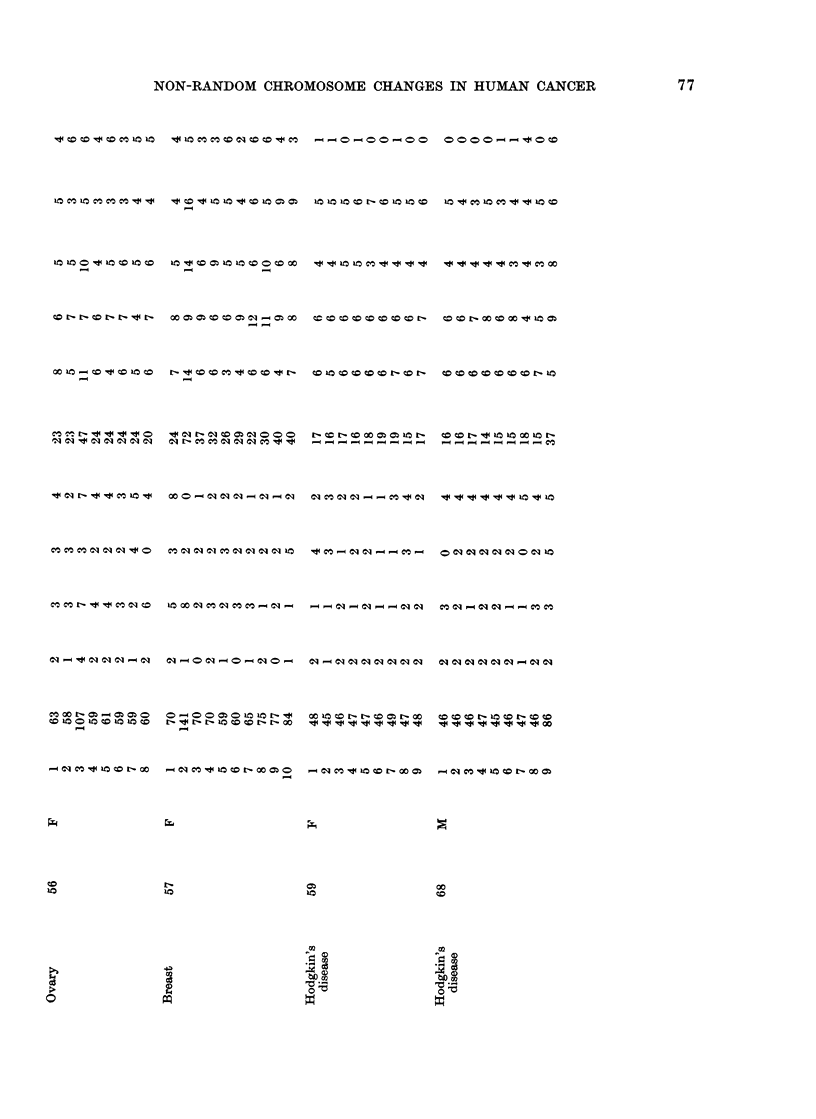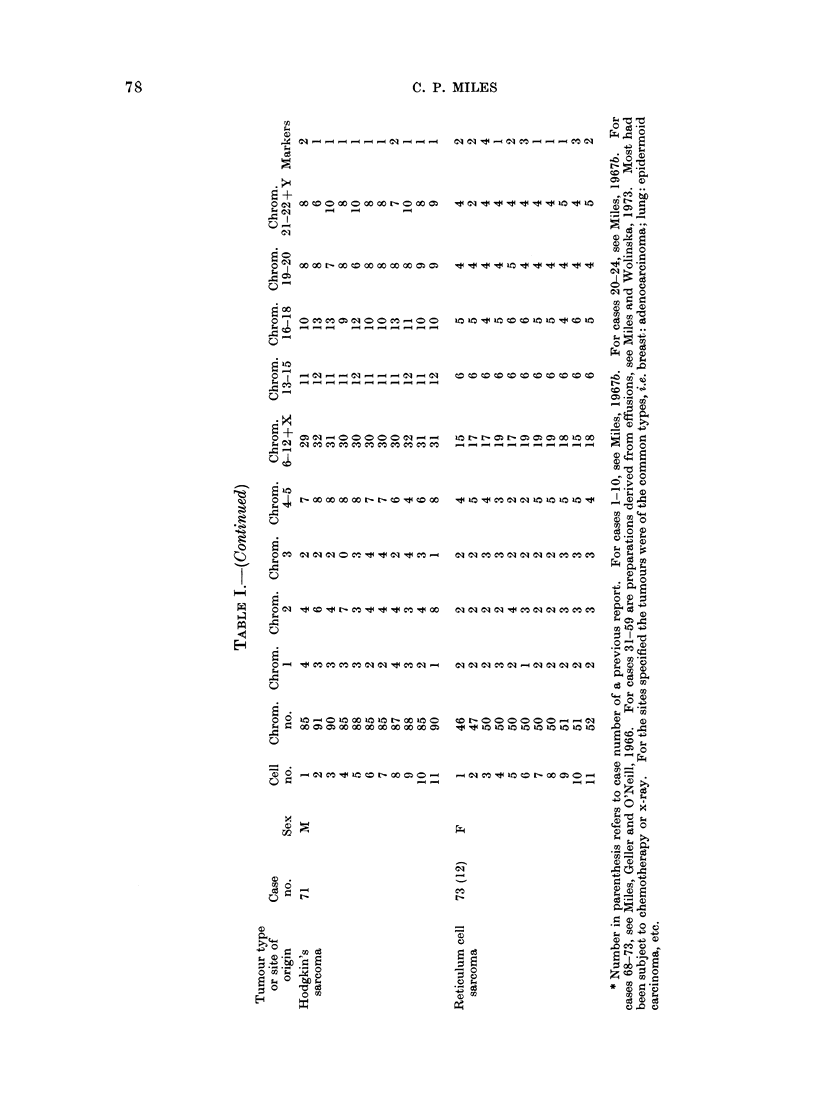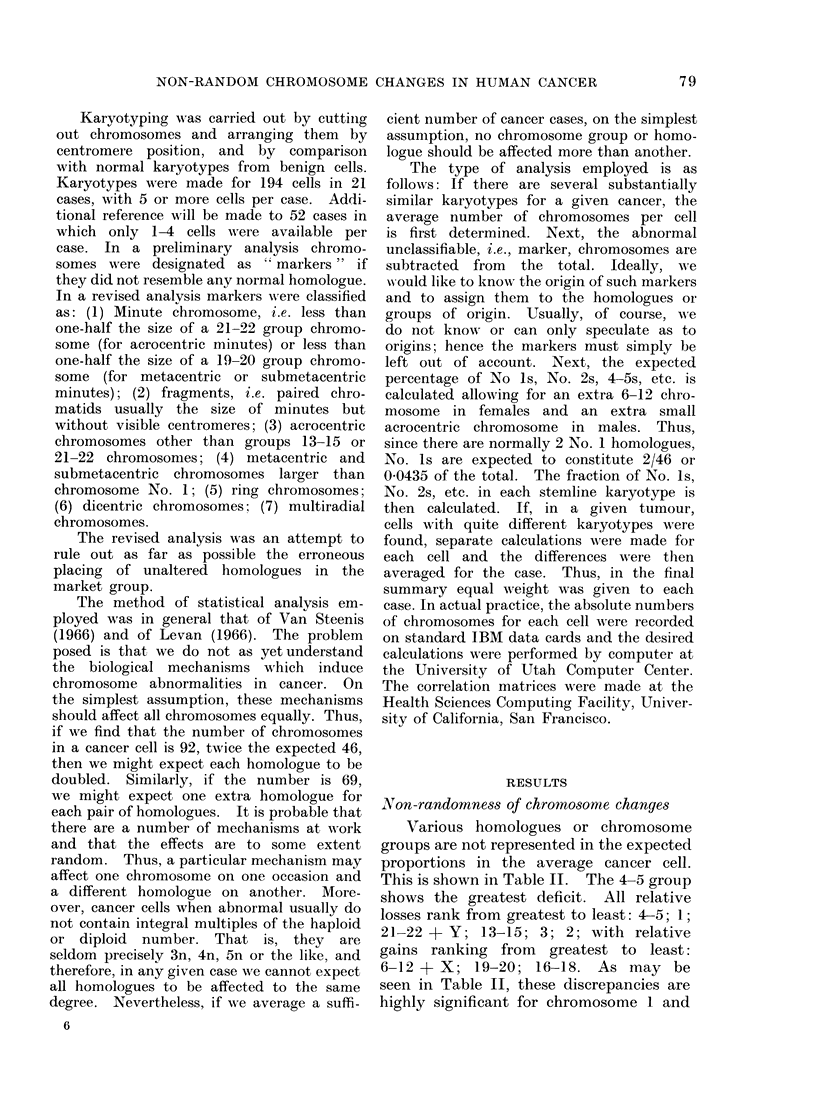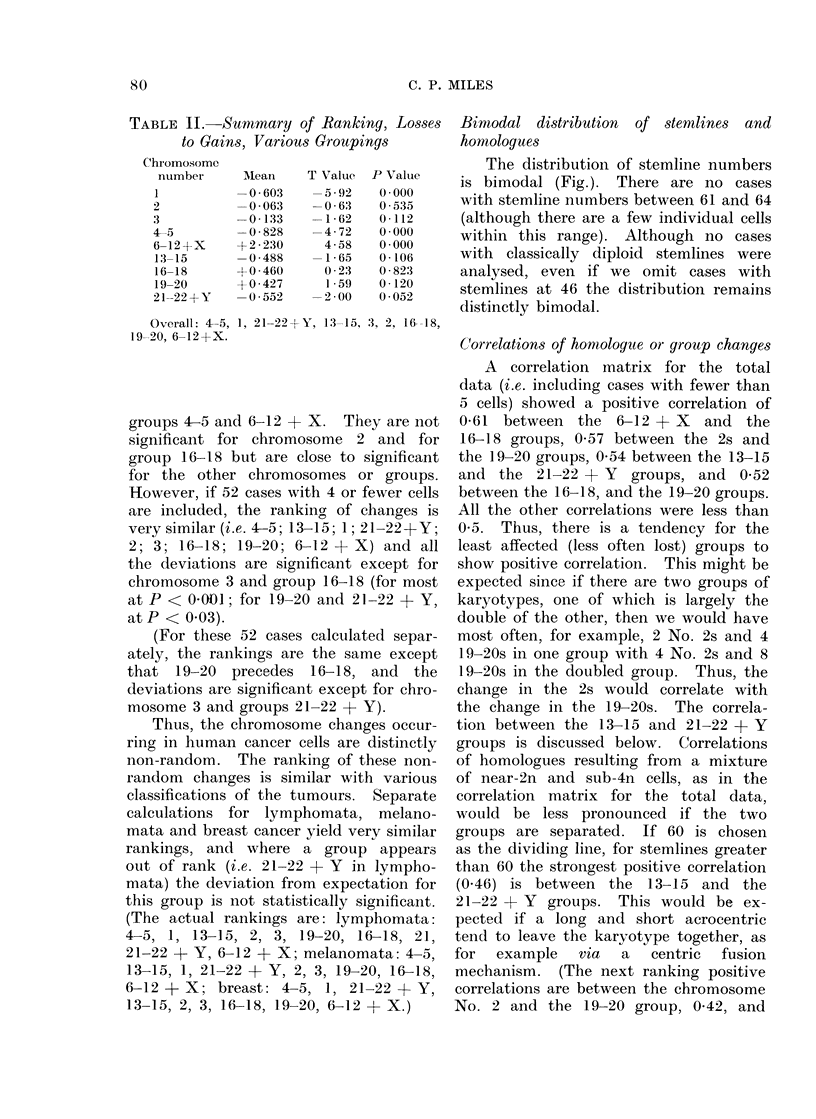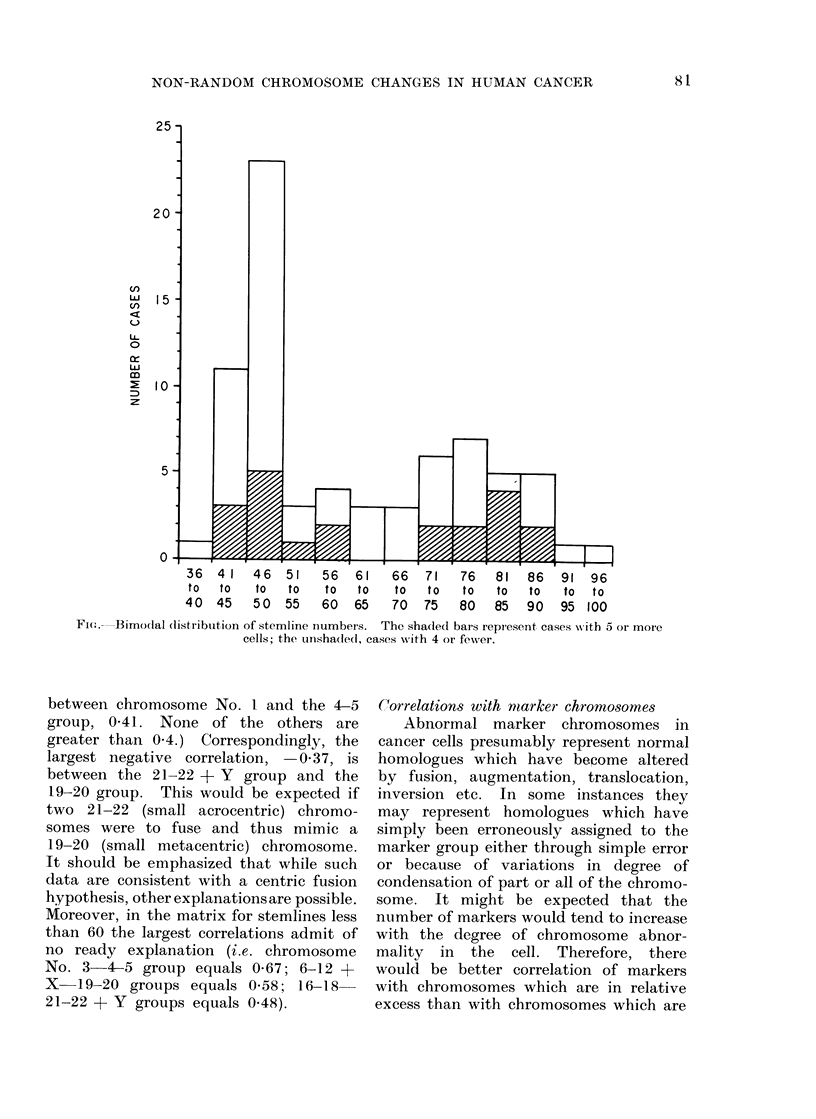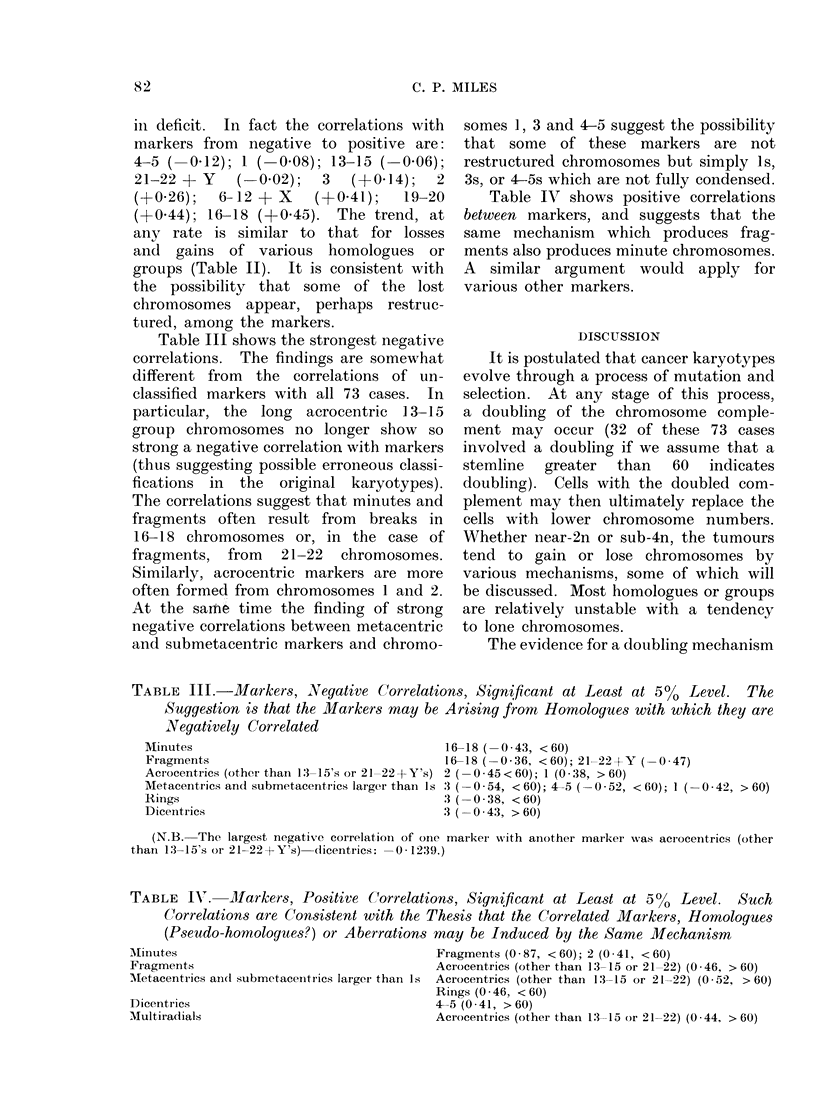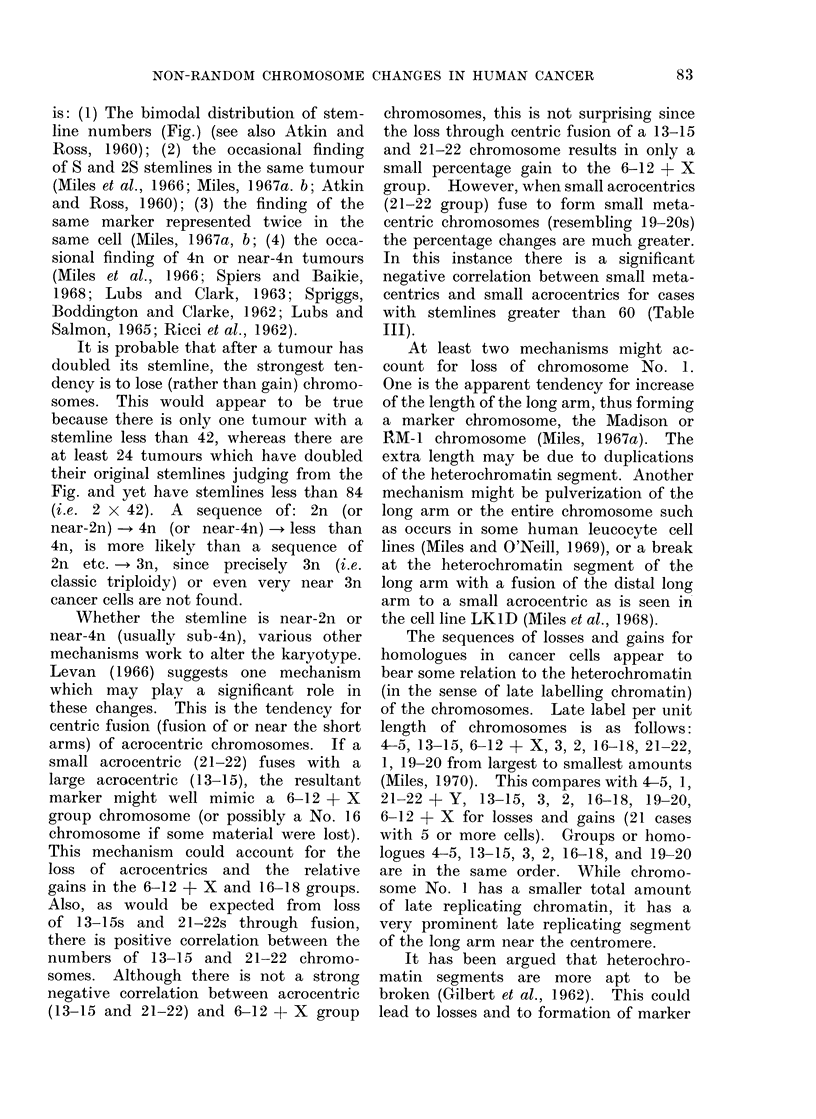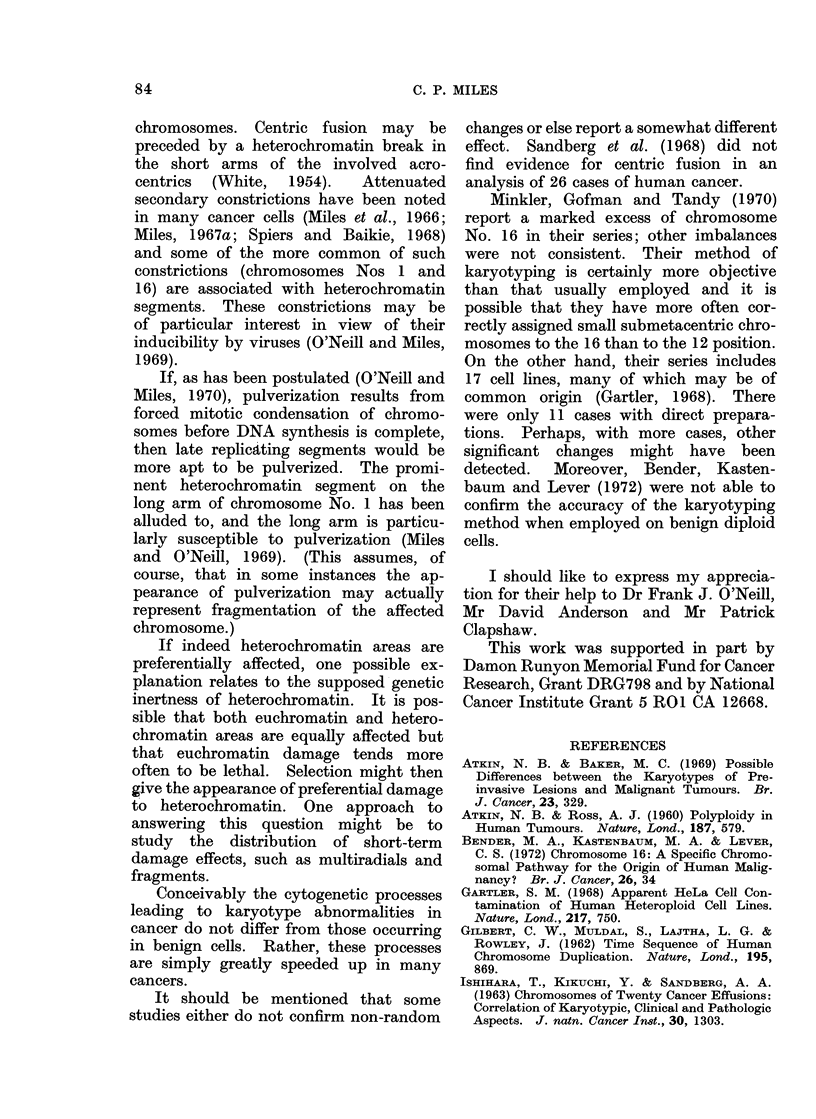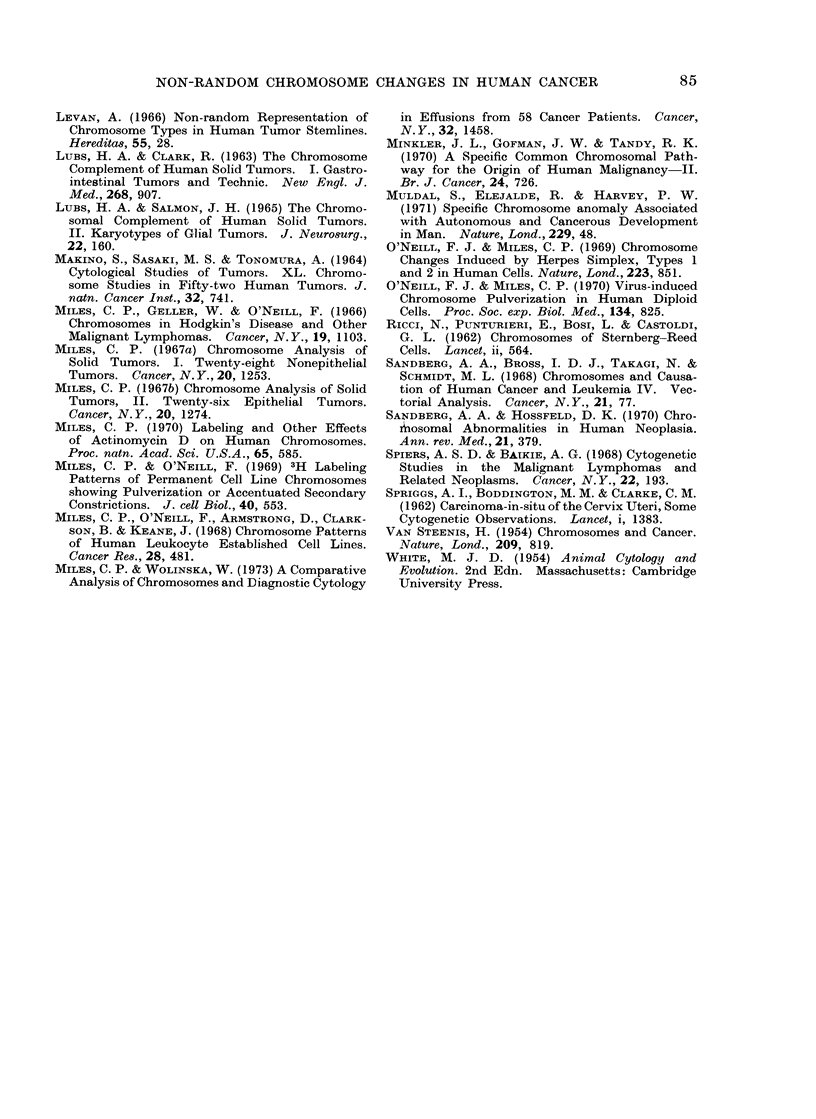# Non-random Chromosome Changes in Human Cancer

**DOI:** 10.1038/bjc.1974.115

**Published:** 1974-07

**Authors:** C. P. Miles

## Abstract

Chromosome changes in human cancer cells appear to evolve by non-random losses and/or gains of particular homologues or groups. It is probable that some of the apparent losses or gains actually represent formation of new chromosome structures, which are then classified as markers or are misclassified as normal homologues. In many cancers these changes appear to continue at a high rate throughout the life of the cancer (so that in some cancers almost every cell will exhibit a different karyotype). In other cancers the rate of change may be slow or arrested so that all cells will have the same abnormal karyotype. One very common step in karyotype evolution is doubling of the entire chromosome complement (2n → 4n or more commonly, S → 2S where S is the stemline number). The 2S cells tend to replace the original stemline. Homologues which have larger amounts of concentrated blocks of heterochromatin (*i.e.* late replicating DNA) seem more apt to be lost.


Br. J. Cancer (1974) 30, 73

### NON-RANDOM CHROMOSOME CHANGES IN HUMAN CANCER

C. P. MILES

*From the Departments of Pathology, University of Utah, Salt Lake City, and Memorial Hospital, Sloan-Kettering Institution, New York City, New York*

Received 30 April 1973. Accepted 8 April 1974

**Summary.—Chromosome changes in human cancer cells appear to evolve by non-random losses and/or gains of particular homologues or groups. It is probable that some of the apparent losses or gains actually represent formation of new chromosome structures, which are then classified as markers or are misclassified as normal homologues. In many cancers these changes appear to continue at a high rate throughout the life of the cancer (so that in some cancers almost every cell will exhibit a different karyotype). In other cancers the rate of change may be slow or arrested so that all cells will have the same abnormal karyotype. One very common step in karyotype evolution is doubling of the entire chromosome complement (2n → 4n or more commonly, S → 2S where S is the stemline number). The 2S cells tend to replace the original stemline. Homologues which have larger amounts of concentrated blocks of heterochromatin (*i.e.* late replicating DNA) seem more apt to be lost.**

So FAR no single feature has been found which distinguishes human cancer cells from benign cells. However, a large majority of human cancers are characterized by gross chromosome abnormalities. The resultant karyotype abnormalities are far more unbalanced than are those which occur in benign somatic cells. Indeed, one may plausibly suspect that were such changes to occur in benign cells, they would prove lethal to these cells. Thus, the capacity of cancer cells so affected to survive may be a peculiarity of cancer. An understanding of the evolution of such changes and of the mechanisms which produce them may help to elucidate the nature of the oncogenic state. Van Steenis (1966) and Levan (1966) have published statistical analyses based respectively on 26 and 40 cases originally described by Makino, Sasaki and Tonomura (1964) and Ishihara, Kikuchi and Sandberg (1963). Atkin and Baker (1969) have reported a similar analysis of a different series. In essence, these authors found that the chromosome changes in human cancer were not random. Rather, some homologues or groups of chromosomes tended to undergo losses, *i.e.*, the acrocentric chromosomes (13–15 and 21–22 + Y) and the B(4–5) group, while some others tended to be augmented, particularly the 6–12 + X group. Muldal, Elejalde and Harvey (1971) have reported similar findings for 5 cases. The following report analyses karyotypes on 21 additional cases originally reported elsewhere.

### MATERIALS AND METHODS

Table I lists cases and karyotypes. All of these analyses were based on direct chromosome preparations, without *in vitro* culture. Seven of the analyses were of solid tumours, 6 primary and 1 metastatic (or recurrent); 14 analyses were of cancer cells in effusion. Only karyotypes with chromosome abnormalities were analysed (thus, tumours with presumed normal stemline karyotypes were omitted). Descriptions of some of these karyotypes have been reported previously (Miles, Geller and O'Neill, 1966; Miles, 1967*a*, *b*; Miles and Wolinska, 1973).

TABLE I.—*Tumour Type or Site of Origin and Chromosome Distribution*

| Tumour type or site of origin | Case no. | Sex | Cell no. | Chrom. no. | Chrom. 1 | Chrom. 2 | Chrom. 3 | Chrom. 4–5 | Chrom. 6–12+X | Chrom. 13–15 | Chrom. 16–18 | Chrom. 19–20 | Chrom. 12–22+Y | Markers |
|---|---|---|---|---|---|---|---|---|---|---|---|---|---|---|
| Reticulum cell sarcoma | 1 (3) | M | 1–5 | 47 | 2 | 2 | 2 | 4 | 16 | 6 | 6 | 4 | 5 | 0 |
| | | | 6 | 48 | 2 | 2 | 2 | 4 | 15 | 6 | 7 | 4 | 6 | 0 |
| | | | 7 | 47 | 2 | 2 | 2 | 4 | 15 | 6 | 6 | 4 | 5 | 1 |
| | | | 8–12 | 47 | 2 | 2 | 2 | 4 | 16 | 6 | 6 | 4 | 5 | 0 |
| | | | 13 | 48 | 2 | 3 | 2 | 4 | 16 | 6 | 6 | 4 | 5 | 0 |
| | | | 14–15 | 47 | 2 | 2 | 2 | 4 | 16 | 6 | 6 | 4 | 5 | 0 |
| Reticulum cell sarcoma | 5 (4) | F | 1–5 | 47 | 1 | 2 | 2 | 4 | 16 | 6 | 7 | 4 | 4 | 1 |
| Melanoma | 8 (16) | F | 1–2 | 42 | 1 | 1 | 2 | 4 | 14 | 6 | 6 | 5 | 2 | 1 |
| | | | 3 | 42 | 1 | 1 | 2 | 4 | 15 | 6 | 6 | 5 | 2 | 0 |
| | | | 4 | 42 | 2 | 2 | 1 | 4 | 14 | 6 | 5 | 4 | 3 | 1 |
| | | | 5 | 42 | 1 | 1 | 2 | 4 | 13 | 6 | 5 | 5 | 4 | 1 |
| | | | 6 | 42 | 1 | 2 | 0 | 4 | 15 | 6 | 6 | 5 | 2 | 1 |
| | | | 7 | 42 | 1 | 2 | 1 | 4 | 14 | 6 | 6 | 5 | 2 | 1 |
| Melanoma | 9 (17) | M | 1 | 77 | 3 | 4 | 3 | 7 | 27 | 8 | 11 | 3 | 10 | 1 |
| | | | 2 | 76 | 4 | 3 | 2 | 7 | 29 | 8 | 7 | 5 | 10 | 1 |
| | | | 3 | 78 | 3 | 3 | 2 | 5 | 30 | 9 | 12 | 3 | 10 | 1 |
| | | | 4 | 77 | 3 | 3 | 3 | 6 | 26 | 8 | 12 | 4 | 10 | 2 |
| | | | 5 | 75 | 3 | 5 | 2 | 4 | 26 | 8 | 10 | 3 | 10 | 4 |
| | | | 6 | 76 | 3 | 5 | 3 | 6 | 27 | 7 | 12 | 3 | 9 | 1 |
| Melanoma | 10 (18) | M | 1 | 72 | 2 | 4 | 4 | 1 | 20 | 10 | 12 | 6 | 9 | 4 |
| | | | 2 | 81 | 3 | 1 | 1 | 2 | 36 | 7 | 10 | 7 | 8 | 6 |
| | | | 3 | 74 | 1 | 2 | 3 | 2 | 31 | 6 | 8 | 6 | 13 | 2 |
| | | | 4 | 71 | 4 | 2 | 2 | 4 | 20 | 9 | 15 | 6 | 7 | 2 |
| | | | 5 | 76 | 1 | 2 | 2 | 4 | 25 | 7 | 11 | 9 | 14 | 1 |
| Gastric | 20 (9) | F | 1 | 81 | 2 | 4 | 3 | 4 | 30 | 8 | 13 | 12 | 1 | 4 |
| | | | 2 | 80 | 2 | 4 | 5 | 5 | 23 | 8 | 12 | 17 | 0 | 4 |
| | | | 3 | 78 | 3 | 5 | 5 | 5 | 25 | 8 | 10 | 15 | 0 | 2 |
| | | | 4 | 81 | 2 | 4 | 3 | 4 | 33 | 10 | 10 | 12 | 0 | 3 |
| | | | 5 | 79 | 3 | 4 | 5 | 6 | 26 | 10 | 17 | 7 | 1 | 0 |
| | | | 6 | 83 | 3 | 4 | 5 | 5 | 29 | 10 | 13 | 13 | 0 | 1 |
| | | | 7 | 85 | 3 | 4 | 6 | 3 | 29 | 10 | 12 | 17 | 0 | 1 |
| | | | 8 | 82 | 3 | 4 | 5 | 5 | 26 | 11 | 14 | 14 | 0 | 0 |
| Colon | 24 (17) | F | 1 | 44 | 2 | 2 | 3 | 2 | 17 | 5 | 7 | 3 | 2 | 1 |
| | | | 2 | 43 | 2 | 2 | 3 | 2 | 15 | 5 | 5 | 4 | 3 | 2 |
| | | | 3 | 43 | 2 | 2 | 1 | 2 | 18 | 5 | 4 | 4 | 4 | 1 |
| | | | 4 | 42 | 2 | 2 | 2 | 2 | 18 | 5 | 4 | 3 | 3 | 1 |
| | | | 5 | 44 | 2 | 2 | 3 | 3 | 16 | 5 | 6 | 4 | 2 | 1 |
| | | | 6 | 43 | 2 | 2 | 2 | 3 | 16 | 5 | 6 | 3 | 3 | 1 |

| | | | | | | | | | | | | | | |
|---|---|---|---|---|---|---|---|---|---|---|---|---|---|---|
| Breast | 31 | F | 1 | 72 | 1 | 2 | 3 | 4 | 29 | 2 | 7 | 9 | 5 | 10 |
| | | | 2 | 72 | 1 | 2 | 0 | 4 | 39 | 2 | 6 | 9 | 5 | 4 |
| | | | 3 | 72 | 1 | 4 | 1 | 4 | 31 | 2 | 6 | 9 | 4 | 10 |
| | | | 4 | 73 | 1 | 3 | 0 | 2 | 38 | 2 | 7 | 10 | 5 | 5 |
| | | | 5 | 73 | 1 | 3 | 0 | 4 | 33 | 2 | 12 | 10 | 5 | 3 |
| | | | 6 | 74 | 1 | 2 | 3 | 4 | 34 | 2 | 6 | 9 | 5 | 8 |
| | | | 7 | 74 | 1 | 2 | 3 | 5 | 31 | 2 | 7 | 9 | 5 | 9 |
| | | | 8 | 74 | 1 | 2 | 4 | 4 | 32 | 2 | 10 | 8 | 6 | 5 |
| Breast | 32 | F | 1 | 47 | 2 | 2 | 2 | 4 | 15 | 5 | 6 | 3 | 4 | 4 |
| | | | 2 | 84 | 1 | 2 | 3 | 4 | 26 | 16 | 9 | 13 | 3 | 7 |
| | | | 3 | 81 | 1 | 5 | 1 | 5 | 20 | 16 | 10 | 10 | 5 | 8 |
| | | | 4 | 81 | 1 | 2 | 5 | 4 | 25 | 16 | 5 | 10 | 4 | 9 |
| | | | 5 | 79 | 1 | 2 | 4 | 4 | 20 | 13 | 10 | 9 | 7 | 9 |
| | | | 6 | 78 | 2 | 5 | 3 | 3 | 26 | 9 | 6 | 9 | 5 | 10 |
| | | | 7 | 70 | 1 | 4 | 3 | 5 | 18 | 7 | 7 | 7 | 4 | 14 |
| | | | 8 | 81 | 1 | 3 | 2 | 5 | 23 | 16 | 8 | 9 | 5 | 9 |
| | | | 9 | 78 | 1 | 2 | 5 | 5 | 28 | 12 | 6 | 7 | 5 | 7 |
| | | | 10 | 85 | 1 | 4 | 3 | 3 | 25 | 13 | 8 | 10 | 4 | 14 |
| | | | 11 | 79 | 1 | 2 | 5 | 4 | 21 | 17 | 7 | 10 | 5 | 7 |
| | | | 12 | 78 | 1 | 3 | 5 | 4 | 24 | 10 | 9 | 9 | 7 | 6 |
| Breast | 36 | F | 1 | 50 | 1 | 2 | 2 | 3 | 20 | 6 | 5 | 6 | 3 | 2 |
| | | | 2 | 48 | 1 | 2 | 2 | 4 | 15 | 6 | 8 | 6 | 3 | 1 |
| | | | 3 | 48 | 1 | 2 | 2 | 5 | 18 | 5 | 5 | 6 | 3 | 1 |
| | | | 4 | 48 | 1 | 2 | 2 | 4 | 18 | 7 | 4 | 6 | 3 | 1 |
| | | | 5 | 47 | 0 | 2 | 1 | 4 | 19 | 6 | 6 | 5 | 3 | 1 |
| Lung | 42 | F | 1 | 56 | 2 | 1 | 2 | 4 | 25 | 5 | 6 | 5 | 5 | 1 |
| | | | 2 | 56 | 2 | 1 | 2 | 5 | 24 | 5 | 6 | 5 | 5 | 1 |
| | | | 3 | 56 | 0 | 2 | 3 | 3 | 25 | 5 | 7 | 5 | 5 | 1 |
| | | | 4 | 56 | 2 | 1 | 2 | 4 | 23 | 5 | 7 | 5 | 5 | 2 |
| | | | 5 | 55 | 2 | 1 | 3 | 4 | 23 | 4 | 6 | 5 | 5 | 2 |
| | | | 6 | 56 | 2 | 1 | 2 | 4 | 26 | 5 | 5 | 5 | 5 | 1 |
| | | | 7 | 57 | 2 | 1 | 2 | 5 | 25 | 5 | 6 | 5 | 5 | 1 |
| | | | 8 | 57 | 2 | 1 | 1 | 4 | 26 | 5 | 7 | 5 | 5 | 1 |
| | | | 9 | 57 | 1 | 1 | 2 | 4 | 27 | 4 | 7 | 5 | 5 | 1 |
| | | | 10 | 56 | 2 | 1 | 2 | 4 | 26 | 5 | 6 | 5 | 5 | 0 |
| Gastric | 44 | F | 1 | 56 | 1 | 2 | 2 | 4 | 20 | 6 | 6 | 5 | 4 | 6 |
| | | | 2 | 47 | 2 | 2 | 2 | 4 | 16 | 7 | 5 | 4 | 5 | 0 |
| | | | 3 | 57 | 0 | 2 | 5 | 4 | 19 | 7 | 7 | 4 | 4 | 5 |
| | | | 4 | 61 | 2 | 4 | 2 | 4 | 26 | 8 | 4 | 5 | 5 | 2 |
| | | | 5 | 77 | 2 | 4 | 4 | 4 | 29 | 9 | 8 | 5 | 6 | 7 |
| | | | 6 | 80 | 1 | 3 | 4 | 2 | 37 | 7 | 9 | 6 | 6 | 5 |
| | | | 7 | 79 | 2 | 3 | 2 | 5 | 35 | 10 | 3 | 7 | 6 | 6 |
| | | | 8 | 80 | 2 | 5 | 2 | 3 | 36 | 8 | 6 | 6 | 6 | 6 |
| | | | 9 | 78 | 0 | 3 | 2 | 5 | 38 | 10 | 5 | 5 | 6 | 4 |
| | | | 10 | 80 | 1 | 3 | 4 | 4 | 36 | 7 | 6 | 6 | 6 | 7 |
| | | | 11 | 80 | 2 | 3 | 2 | 4 | 36 | 9 | 7 | 5 | 6 | 6 |
| | | | 12 | 84 | 3 | 3 | 0 | 5 | 36 | 9 | 11 | 5 | 6 | 6 |
| | | | 13 | 88 | 1 | 4 | 2 | 4 | 36 | 10 | 9 | 7 | 6 | 9 |
| | | | 14 | 81 | 2 | 4 | 2 | 3 | 40 | 8 | 5 | 5 | 6 | 6 |

TABLE I.—(*Continued*)

| Tumour type or site of origin | Case no. | Sex | Cell no. | Chrom. no. | Chrom. 1 | Chrom. 2 | Chrom. 3 | Chrom. 4–5 | Chrom. 6–12+X | Chrom. 13–15 | Chrom. 16–18 | Chrom. 19–20 | Chrom. 21–22+Y | Markers |
|---|---|---|---|---|---|---|---|---|---|---|---|---|---|---|
| Breast | 48 | F | 1 | 73 | 0 | 2 | 2 | 3 | 27 | 7 | 12 | 4 | 0 | 16 |
| | | | 2 | 137 | 2 | 3 | 2 | 6 | 66 | 15 | 22 | 7 | 0 | 14 |
| | | | 3 | 68 | 2 | 1 | 0 | 0 | 32 | 9 | 13 | 4 | 1 | 6 |
| | | | 4 | 60 | 2 | 1 | 3 | 4 | 15 | 11 | 6 | 7 | 3 | 8 |
| | | | 5 | 54 | 3 | 2 | 1 | 5 | 17 | 6 | 9 | 4 | 1 | 6 |
| | | | 6 | 65 | 0 | 1 | 1 | 1 | 22 | 7 | 11 | 7 | 1 | 14 |
| | | | 7 | 69 | 1 | 2 | 2 | 5 | 23 | 7 | 11 | 12 | 0 | 6 |
| | | | 8 | 135 | 3 | 3 | 3 | 5 | 48 | 11 | 19 | 14 | 3 | 26 |
| | | | 9 | 72 | 0 | 2 | 4 | 2 | 34 | 12 | 8 | 3 | 2 | 5 |
| | | | 10 | 74 | 1 | 6 | 2 | 5 | 26 | 6 | 12 | 4 | 2 | 10 |
| | | | 11 | 76 | 1 | 1 | 1 | 5 | 29 | 6 | 12 | 6 | 4 | 11 |
| | | | 12 | 90 | 1 | 1 | 3 | 4 | 35 | 11 | 13 | 7 | 1 | 14 |
| | | | 13 | 77 | 2 | 3 | 2 | 7 | 23 | 10 | 9 | 5 | 6 | 10 |
| | | | 14 | 72 | 2 | 0 | 3 | 6 | 26 | 10 | 10 | 5 | 2 | 8 |
| | | | 15 | 56 | 1 | 1 | 4 | 4 | 19 | 7 | 10 | 6 | 1 | 3 |
| | | | 16 | 66 | 2 | 0 | 1 | 1 | 24 | 10 | 14 | 5 | 2 | 7 |
| | | | 17 | 61 | 2 | 3 | 2 | 4 | 26 | 5 | 7 | 8 | 0 | 4 |
| | | | 18 | 75 | 3 | 1 | 4 | 4 | 27 | 8 | 14 | 5 | 2 | 7 |
| | | | 19 | 73 | 2 | 4 | 3 | 7 | 29 | 10 | 5 | 4 | 2 | 7 |
| | | | 20 | 71 | 1 | 2 | 3 | 4 | 30 | 10 | 10 | 4 | 2 | 5 |
| | | | 21 | 75 | 2 | 1 | 4 | 3 | 25 | 10 | 10 | 5 | 3 | 12 |
| | | | 22 | 72 | 0 | 3 | 3 | 3 | 27 | 12 | 12 | 4 | 2 | 6 |
| | | | 23 | 71 | 1 | 2 | 3 | 4 | 28 | 8 | 12 | 3 | 2 | 8 |
| | | | 24 | 67 | 0 | 2 | 3 | 3 | 34 | 5 | 6 | 3 | 1 | 10 |
| | | | 25 | 136 | 0 | 1 | 2 | 0 | 59 | 13 | 25 | 16 | 2 | 18 |
| Hodgkin's disease | 52 | F | 1–2 | 46 | 2 | 2 | 2 | 4 | 15 | 6 | 6 | 4 | 4 | 1 |
| | | | 3 | 46 | 2 | 2 | 2 | 3 | 16 | 6 | 6 | 4 | 4 | 1 |
| | | | 4 | 46 | 2 | 2 | 2 | 4 | 15 | 5 | 6 | 4 | 4 | 2 |
| | | | 5 | 47 | 1 | 2 | 3 | 4 | 18 | 5 | 6 | 4 | 4 | 0 |
| Ovary | 53 | F | 1 | 71 | 2 | 3 | 3 | 5 | 27 | 8 | 14 | 4 | 4 | 1 |
| | | | 2 | 66 | 2 | 3 | 3 | 5 | 27 | 9 | 9 | 4 | 2 | 2 |
| | | | 3 | 70 | 2 | 1 | 1 | 4 | 33 | 11 | 8 | 1 | 4 | 5 |
| | | | 4 | 68 | 1 | 1 | 2 | 0 | 32 | 13 | 11 | 4 | 3 | 1 |
| | | | 5 | 72 | 2 | 1 | 3 | 3 | 31 | 8 | 12 | 6 | 3 | 3 |

| | | | | | | | | | | | | | | |
|---|---|---|---|---|---|---|---|---|---|---|---|---|---|---|
| Ovary | 56 | F | 1 | 63 | 2 | 3 | 3 | 4 | 23 | 8 | 6 | 5 | 5 | 4 |
| | | | 2 | 58 | 1 | 3 | 3 | 2 | 23 | 5 | 7 | 5 | 3 | 6 |
| | | | 3 | 107 | 4 | 7 | 3 | 7 | 47 | 11 | 7 | 10 | 5 | 6 |
| | | | 4 | 59 | 2 | 4 | 2 | 4 | 24 | 6 | 6 | 4 | 3 | 4 |
| | | | 5 | 61 | 2 | 4 | 2 | 4 | 24 | 4 | 7 | 5 | 3 | 6 |
| | | | 6 | 59 | 2 | 3 | 2 | 3 | 24 | 6 | 7 | 6 | 3 | 3 |
| | | | 7 | 59 | 1 | 2 | 4 | 5 | 24 | 5 | 4 | 5 | 4 | 5 |
| | | | 8 | 60 | 2 | 6 | 0 | 4 | 20 | 6 | 7 | 6 | 4 | 5 |
| Breast | 57 | F | 1 | 70 | 2 | 5 | 3 | 8 | 24 | 7 | 8 | 5 | 4 | 4 |
| | | | 2 | 141 | 1 | 8 | 2 | 0 | 72 | 14 | 9 | 14 | 16 | 5 |
| | | | 3 | 70 | 0 | 2 | 2 | 1 | 37 | 6 | 9 | 6 | 4 | 3 |
| | | | 4 | 70 | 2 | 3 | 2 | 2 | 32 | 6 | 6 | 9 | 5 | 3 |
| | | | 5 | 59 | 1 | 2 | 3 | 2 | 26 | 3 | 6 | 5 | 5 | 6 |
| | | | 6 | 60 | 0 | 3 | 2 | 2 | 29 | 4 | 9 | 5 | 4 | 2 |
| | | | 7 | 65 | 1 | 3 | 2 | 1 | 22 | 6 | 12 | 6 | 6 | 6 |
| | | | 8 | 75 | 2 | 1 | 2 | 2 | 30 | 6 | 11 | 10 | 5 | 6 |
| | | | 9 | 77 | 0 | 2 | 2 | 1 | 40 | 4 | 9 | 6 | 9 | 4 |
| | | | 10 | 84 | 1 | 1 | 5 | 2 | 40 | 7 | 8 | 8 | 9 | 3 |
| Hodgkin's disease | 59 | F | 1 | 48 | 2 | 1 | 4 | 2 | 17 | 6 | 6 | 4 | 5 | 1 |
| | | | 2 | 45 | 1 | 1 | 3 | 3 | 16 | 5 | 6 | 4 | 5 | 1 |
| | | | 3 | 46 | 2 | 2 | 1 | 2 | 17 | 6 | 6 | 5 | 5 | 0 |
| | | | 4 | 47 | 2 | 1 | 2 | 2 | 16 | 6 | 6 | 5 | 6 | 1 |
| | | | 5 | 47 | 2 | 2 | 2 | 1 | 18 | 6 | 6 | 3 | 7 | 0 |
| | | | 6 | 46 | 2 | 1 | 1 | 1 | 19 | 6 | 6 | 4 | 6 | 0 |
| | | | 7 | 49 | 2 | 1 | 1 | 3 | 19 | 7 | 6 | 4 | 5 | 1 |
| | | | 8 | 47 | 2 | 2 | 3 | 4 | 15 | 6 | 6 | 4 | 5 | 0 |
| | | | 9 | 48 | 2 | 2 | 1 | 2 | 17 | 7 | 7 | 4 | 6 | 0 |
| Hodgkin's disease | 68 | M | 1 | 46 | 2 | 3 | 0 | 4 | 16 | 6 | 6 | 4 | 5 | 0 |
| | | | 2 | 46 | 2 | 2 | 2 | 4 | 16 | 6 | 6 | 4 | 4 | 0 |
| | | | 3 | 46 | 2 | 1 | 2 | 4 | 17 | 6 | 7 | 4 | 3 | 0 |
| | | | 4 | 47 | 2 | 2 | 2 | 4 | 14 | 6 | 8 | 4 | 5 | 0 |
| | | | 5 | 45 | 2 | 2 | 2 | 4 | 15 | 6 | 6 | 4 | 3 | 1 |
| | | | 6 | 46 | 2 | 1 | 2 | 4 | 15 | 6 | 8 | 3 | 4 | 1 |
| | | | 7 | 47 | 1 | 1 | 0 | 5 | 18 | 6 | 4 | 4 | 4 | 4 |
| | | | 8 | 46 | 2 | 3 | 2 | 4 | 15 | 7 | 5 | 3 | 5 | 0 |
| | | | 9 | 86 | 2 | 3 | 5 | 5 | 37 | 5 | 9 | 8 | 6 | 6 |

TABLE I.—(*Continued*)

| Tumour type or site of origin | Case no. | Sex | Cell no. | Chrom. no. | Chrom. 1 | Chrom. 2 | Chrom. 3 | Chrom. 4–5 | Chrom. 6–12+X | Chrom. 13–15 | Chrom. 16–18 | Chrom. 19–20 | Chrom. 21–22+Y | Markers |
|---|---|---|---|---|---|---|---|---|---|---|---|---|---|---|
| Hodgkin's sarcoma | 71 | M | 1 | 85 | 4 | 4 | 2 | 7 | 29 | 11 | 10 | 8 | 8 | 2 |
| | | | 2 | 91 | 3 | 6 | 2 | 8 | 32 | 12 | 13 | 8 | 6 | 1 |
| | | | 3 | 90 | 3 | 4 | 2 | 8 | 31 | 11 | 13 | 7 | 10 | 1 |
| | | | 4 | 85 | 3 | 7 | 0 | 8 | 30 | 11 | 9 | 8 | 8 | 1 |
| | | | 5 | 88 | 3 | 3 | 3 | 8 | 30 | 12 | 12 | 6 | 10 | 1 |
| | | | 6 | 85 | 2 | 4 | 4 | 7 | 30 | 11 | 10 | 8 | 8 | 1 |
| | | | 7 | 85 | 2 | 4 | 4 | 7 | 30 | 11 | 10 | 8 | 8 | 1 |
| | | | 8 | 87 | 4 | 4 | 2 | 6 | 30 | 11 | 13 | 8 | 7 | 2 |
| | | | 9 | 88 | 3 | 3 | 4 | 4 | 32 | 12 | 11 | 8 | 10 | 1 |
| | | | 10 | 85 | 2 | 4 | 3 | 6 | 31 | 11 | 10 | 9 | 8 | 1 |
| | | | 11 | 90 | 1 | 8 | 1 | 8 | 31 | 12 | 10 | 9 | 9 | 1 |
| Reticulum cell sarcoma | 73 (12) | F | 1 | 46 | 2 | 2 | 2 | 4 | 15 | 6 | 5 | 4 | 4 | 2 |
| | | | 2 | 47 | 2 | 2 | 2 | 5 | 17 | 6 | 5 | 4 | 2 | 2 |
| | | | 3 | 50 | 2 | 2 | 3 | 4 | 17 | 6 | 4 | 4 | 4 | 4 |
| | | | 4 | 50 | 3 | 2 | 3 | 3 | 19 | 6 | 5 | 4 | 4 | 1 |
| | | | 5 | 50 | 2 | 4 | 2 | 2 | 17 | 6 | 6 | 5 | 4 | 2 |
| | | | 6 | 50 | 1 | 3 | 2 | 2 | 19 | 6 | 6 | 4 | 4 | 3 |
| | | | 7 | 50 | 2 | 2 | 2 | 5 | 19 | 6 | 5 | 4 | 4 | 1 |
| | | | 8 | 50 | 2 | 2 | 2 | 5 | 19 | 6 | 5 | 4 | 4 | 1 |
| | | | 9 | 51 | 2 | 3 | 3 | 5 | 18 | 6 | 4 | 4 | 5 | 1 |
| | | | 10 | 51 | 2 | 3 | 3 | 5 | 15 | 6 | 6 | 4 | 4 | 3 |
| | | | 11 | 52 | 2 | 3 | 3 | 4 | 18 | 6 | 5 | 4 | 5 | 2 |

* Number in parenthesis refers to case number of a previous report. For cases 1–10, see Miles, 1967*b*. For cases 20–24, see Miles, 1967*b*. For cases 68–73, see Miles, Geller and O'Neill, 1966. For cases 31–59 are preparations derived from effusions, see Miles and Wolinska, 1973. Most had been subject to chemotherapy or x-ray. For the sites specified the tumours were of the common types, *i.e.* breast: adenocarcinoma; lung: epidermoid carcinoma, etc.

Karyotyping was carried out by cutting out chromosomes and arranging them by centromere position, and by comparison with normal karyotypes from benign cells. Karyotypes were made for 194 cells in 21 cases, with 5 or more cells per case. Additional reference will be made to 52 cases in which only 1–4 cells were available per case. In a preliminary analysis chromosomes were designated as "markers" if they did not resemble any normal homologue. In a revised analysis markers were classified as: (1) Minute chromosome, *i.e.* less than one-half the size of a 21–22 group chromosome (for acrocentric minutes) or less than one-half the size of a 19–20 group chromosome (for metacentric or submetacentric minutes); (2) fragments, *i.e.* paired chromatids usually the size of minutes but without visible centromeres; (3) acrocentric chromosomes other than groups 13–15 or 21–22 chromosomes; (4) metacentric and submetacentric chromosomes larger than chromosome No. 1; (5) ring chromosomes; (6) dicentric chromosomes; (7) multiradial chromosomes.

The revised analysis was an attempt to rule out as far as possible the erroneous placing of unaltered homologues in the market group.

The method of statistical analysis employed was in general that of Van Steenis (1966) and of Levan (1966). The problem posed is that we do not as yet understand the biological mechanisms which induce chromosome abnormalities in cancer. On the simplest assumption, these mechanisms should affect all chromosomes equally. Thus, if we find that the number of chromosomes in a cancer cell is 92, twice the expected 46, then we might expect each homologue to be doubled. Similarly, if the number is 69, we might expect one extra homologue for each pair of homologues. It is probable that there are a number of mechanisms at work and that the effects are to some extent random. Thus, a particular mechanism may affect one chromosome on one occasion and a different homologue on another. Moreover, cancer cells when abnormal usually do not contain integral multiples of the haploid or diploid number. That is, they are seldom precisely 3n, 4n, 5n or the like, and therefore, in any given case we cannot expect all homologues to be affected to the same degree. Nevertheless, if we average a sufficient number of cancer cases, on the simplest assumption, no chromosome group or homologue should be affected more than another.

The type of analysis employed is as follows: If there are several substantially similar karyotypes for a given cancer, the average number of chromosomes per cell is first determined. Next, the abnormal unclassifiable, *i.e.*, marker, chromosomes are subtracted from the total. Ideally, we would like to know the origin of such markers and to assign them to the homologues or groups of origin. Usually, of course, we do not know or can only speculate as to origins; hence the markers must simply be left out of account. Next, the expected percentage of No 1s, No. 2s, 4–5s, etc. is calculated allowing for an extra 6–12 chromosome in females and an extra small acrocentric chromosome in males. Thus, since there are normally 2 No. 1 homologues, No. 1s are expected to constitute 2/46 or 0·0435 of the total. The fraction of No. 1s, No. 2s, etc. in each stemline karyotype is then calculated. If, in a given tumour, cells with quite different karyotypes were found, separate calculations were made for each cell and the differences were then averaged for the case. Thus, in the final summary equal weight was given to each case. In actual practice, the absolute numbers of chromosomes for each cell were recorded on standard IBM data cards and the desired calculations were performed by computer at the University of Utah Computer Center. The correlation matrices were made at the Health Sciences Computing Facility, University of California, San Francisco.

### RESULTS

### *Non-randomness of chromosome changes*

Various homologues or chromosome groups are not represented in the expected proportions in the average cancer cell. This is shown in Table II. The 4–5 group shows the greatest deficit. All relative losses rank from greatest to least: 4–5; 1; 21–22 + Y; 13–15; 3; 2; with relative gains ranking from greatest to least: 6–12 + X; 19–20; 16–18. As may be seen in Table II, these discrepancies are highly significant for chromosome 1 and

TABLE II.—*Summary of Ranking, Losses to Gains, Various Groupings*

| Chromosome number | Mean | T Value | *P* Value |
|---|---|---|---|
| 1 | −0·603 | −5·92 | 0·000 |
| 2 | −0·063 | −0·63 | 0·535 |
| 3 | −0·133 | −1·62 | 0·112 |
| 4–5 | −0·828 | −4·72 | 0·000 |
| 6–12+X | +2·230 | 4·58 | 0·000 |
| 13–15 | −0·488 | −1·65 | 0·106 |
| 16–18 | +0·460 | 0·23 | 0·823 |
| 19–20 | +0·427 | 1·59 | 0·120 |
| 21–22+Y | −0·552 | −2·00 | 0·052 |

Overall: 4–5, 1, 21–22+Y, 13–15, 3, 2, 16–18, 19–20, 6–12+X.

groups 4–5 and 6–12 + X. They are not significant for chromosome 2 and for group 16–18 but are close to significant for the other chromosomes or groups. However, if 52 cases with 4 or fewer cells are included, the ranking of changes is very similar (*i.e.* 4–5; 13–15; 1; 21–22+Y; 2; 3; 16–18; 19–20; 6–12 + X) and all the deviations are significant except for chromosome 3 and group 16–18 (for most at $P < 0·001$; for 19–20 and 21–22 + Y, at $P < 0·03$).

(For these 52 cases calculated separately, the rankings are the same except that 19–20 precedes 16–18, and the deviations are significant except for chromosome 3 and groups 21–22 + Y).

Thus, the chromosome changes occurring in human cancer cells are distinctly non-random. The ranking of these non-random changes is similar with various classifications of the tumours. Separate calculations for lymphomata, melanomata and breast cancer yield very similar rankings, and where a group appears out of rank (*i.e.* 21–22 + Y in lymphomata) the deviation from expectation for this group is not statistically significant. (The actual rankings are: lymphomata: 4–5, 1, 13–15, 2, 3, 19–20, 16–18, 21, 21–22 + Y, 6–12 + X; melanomata: 4–5, 13–15, 1, 21–22 + Y, 2, 3, 19–20, 16–18, 6–12 + X; breast: 4–5, 1, 21–22 + Y, 13–15, 2, 3, 16–18, 19–20, 6–12 + X.)

*Bimodal distribution of stemlines and homologues*

The distribution of stemline numbers is bimodal (Fig.). There are no cases with stemline numbers between 61 and 64 (although there are a few individual cells within this range). Although no cases with classically diploid stemlines were analysed, even if we omit cases with stemlines at 46 the distribution remains distinctly bimodal.

*Correlations of homologue or group changes*

A correlation matrix for the total data (*i.e.* including cases with fewer than 5 cells) showed a positive correlation of 0·61 between the 6–12 + X and the 16–18 groups, 0·57 between the 2s and the 19–20 groups, 0·54 between the 13–15 and the 21–22 + Y groups, and 0·52 between the 16–18, and the 19–20 groups. All the other correlations were less than 0·5. Thus, there is a tendency for the least affected (less often lost) groups to show positive correlation. This might be expected since if there are two groups of karyotypes, one of which is largely the double of the other, then we would have most often, for example, 2 No. 2s and 4 19–20s in one group with 4 No. 2s and 8 19–20s in the doubled group. Thus, the change in the 2s would correlate with the change in the 19–20s. The correlation between the 13–15 and 21–22 + Y groups is discussed below. Correlations of homologues resulting from a mixture of near-2n and sub-4n cells, as in the correlation matrix for the total data, would be less pronounced if the two groups are separated. If 60 is chosen as the dividing line, for stemlines greater than 60 the strongest positive correlation (0·46) is between the 13–15 and the 21–22 + Y groups. This would be expected if a long and short acrocentric tend to leave the karyotype together, as for example *via* a centric fusion mechanism. (The next ranking positive correlations are between the chromosome No. 2 and the 19–20 group, 0·42, and

FIG.—Bimodal distribution of stemline numbers. The shaded bars represent cases with 5 or more cells; the unshaded, cases with 4 or fewer.

between chromosome No. 1 and the 4–5 group, 0·41. None of the others are greater than 0·4.) Correspondingly, the largest negative correlation, —0·37, is between the 21–22 + Y group and the 19–20 group. This would be expected if two 21–22 (small acrocentric) chromosomes were to fuse and thus mimic a 19–20 (small metacentric) chromosome. It should be emphasized that while such data are consistent with a centric fusion hypothesis, other explanations are possible. Moreover, in the matrix for stemlines less than 60 the largest correlations admit of no ready explanation (*i.e.* chromosome No. 3—4–5 group equals 0·67; 6–12 + X—19–20 groups equals 0·58; 16–18—21–22 + Y groups equals 0·48).

*Correlations with marker chromosomes*

Abnormal marker chromosomes in cancer cells presumably represent normal homologues which have become altered by fusion, augmentation, translocation, inversion etc. In some instances they may represent homologues which have simply been erroneously assigned to the marker group either through simple error or because of variations in degree of condensation of part or all of the chromosome. It might be expected that the number of markers would tend to increase with the degree of chromosome abnormality in the cell. Therefore, there would be better correlation of markers with chromosomes which are in relative excess than with chromosomes which are in deficit. In fact the correlations with markers from negative to positive are: 4–5 (−0·12); 1 (−0·08); 13–15 (−0·06); 21–22 + Y (−0·02); 3 (+0·14); 2 (+0·26); 6-12 + X (+0·41); 19–20 (+0·44); 16–18 (+0·45). The trend, at any rate is similar to that for losses and gains of various homologues or groups (Table II). It is consistent with the possibility that some of the lost chromosomes appear, perhaps restructured, among the markers.

Table III shows the strongest negative correlations. The findings are somewhat different from the correlations of unclassified markers with all 73 cases. In particular, the long acrocentric 13–15 group chromosomes no longer show so strong a negative correlation with markers (thus suggesting possible erroneous classifications in the original karyotypes). The correlations suggest that minutes and fragments often result from breaks in 16–18 chromosomes or, in the case of fragments, from 21–22 chromosomes. Similarly, acrocentric markers are more often formed from chromosomes 1 and 2. At the same time the finding of strong negative correlations between metacentric and submetacentric markers and chromosomes 1, 3 and 4–5 suggest the possibility that some of these markers are not restructured chromosomes but simply 1s, 3s, or 4–5s which are not fully condensed.

Table IV shows positive correlations *between* markers, and suggests that the same mechanism which produces fragments also produces minute chromosomes. A similar argument would apply for various other markers.

### DISCUSSION

It is postulated that cancer karyotypes evolve through a process of mutation and selection. At any stage of this process, a doubling of the chromosome complement may occur (32 of these 73 cases involved a doubling if we assume that a stemline greater than 60 indicates doubling). Cells with the doubled complement may then ultimately replace the cells with lower chromosome numbers. Whether near-2n or sub-4n, the tumours tend to gain or lose chromosomes by various mechanisms, some of which will be discussed. Most homologues or groups are relatively unstable with a tendency to lone chromosomes.

The evidence for a doubling mechanism

TABLE III.—*Markers, Negative Correlations, Significant at Least at 5% Level. The Suggestion is that the Markers may be Arising from Homologues with which they are Negatively Correlated*

| | |
|---|---|
| Minutes | 16–18 (−0·43, <60) |
| Fragments | 16–18 (−0·36, <60); 21–22+Y (−0·47) |
| Acrocentrics (other than 13–15's or 21–22+Y's) | 2 (−0·45<60); 1 (0·38, >60) |
| Metacentrics and submetacentrics larger than 1s | 3 (−0·54, <60); 4–5 (−0·52, <60); 1 (−0·42, >60) |
| Rings | 3 (−0·38, <60) |
| Dicentrics | 3 (−0·43, >60) |

(N.B.—The largest negative correlation of one marker with another marker was acrocentrics (other than 13–15's or 21–22+Y's)—dicentrics: −0·1239.)

TABLE IV.—*Markers, Positive Correlations, Significant at Least at 5% Level. Such Correlations are Consistent with the Thesis that the Correlated Markers, Homologues (Pseudo-homologues?) or Aberrations may be Induced by the Same Mechanism*

| | |
|---|---|
| Minutes | Fragments (0·87, <60); 2 (0·41, <60) |
| Fragments | Acrocentrics (other than 13–15 or 21–22) (0·46, >60) |
| Metacentrics and submetacentrics larger than 1s | Acrocentrics (other than 13–15 or 21–22) (0·52, >60) |
| | Rings (0·46, <60) |
| Dicentrics | 4–5 (0·41, >60) |
| Multiradials | Acrocentrics (other than 13–15 or 21–22) (0·44, >60) |

is: (1) The bimodal distribution of stemline numbers (Fig.) (see also Atkin and Ross, 1960); (2) the occasional finding of S and 2S stemlines in the same tumour (Miles *et al.*, 1966; Miles, 1967*a*. *b*; Atkin and Ross, 1960); (3) the finding of the same marker represented twice in the same cell (Miles, 1967*a*, *b*; (4) the occasional finding of 4n or near-4n tumours (Miles *et al.*, 1966; Spiers and Baikie, 1968; Lubs and Clark, 1963; Spriggs, Boddington and Clarke, 1962; Lubs and Salmon, 1965; Ricci *et al.*, 1962).

It is probable that after a tumour has doubled its stemline, the strongest tendency is to lose (rather than gain) chromosomes. This would appear to be true because there is only one tumour with a stemline less than 42, whereas there are at least 24 tumours which have doubled their original stemlines judging from the Fig. and yet have stemlines less than 84 (*i.e.* 2 × 42). A sequence of: 2n (or near-2n) → 4n (or near-4n) → less than 4n, is more likely than a sequence of 2n etc. → 3n, since precisely 3n (*i.e.* classic triploidy) or even very near 3n cancer cells are not found.

Whether the stemline is near-2n or near-4n (usually sub-4n), various other mechanisms work to alter the karyotype. Levan (1966) suggests one mechanism which may play a significant role in these changes. This is the tendency for centric fusion (fusion of or near the short arms) of acrocentric chromosomes. If a small acrocentric (21–22) fuses with a large acrocentric (13–15), the resultant marker might well mimic a 6–12 + X group chromosome (or possibly a No. 16 chromosome if some material were lost). This mechanism could account for the loss of acrocentrics and the relative gains in the 6–12 + X and 16–18 groups. Also, as would be expected from loss of 13–15s and 21–22s through fusion, there is positive correlation between the numbers of 13–15 and 21–22 chromosomes. Although there is not a strong negative correlation between acrocentric (13–15 and 21–22) and 6–12 + X group chromosomes, this is not surprising since the loss through centric fusion of a 13–15 and 21–22 chromosome results in only a small percentage gain to the 6–12 + X group. However, when small acrocentrics (21–22 group) fuse to form small metacentric chromosomes (resembling 19–20s) the percentage changes are much greater. In this instance there is a significant negative correlation between small metacentrics and small acrocentrics for cases with stemlines greater than 60 (Table III).

At least two mechanisms might account for loss of chromosome No. 1. One is the apparent tendency for increase of the length of the long arm, thus forming a marker chromosome, the Madison or RM-1 chromosome (Miles, 1967*a*). The extra length may be due to duplications of the heterochromatin segment. Another mechanism might be pulverization of the long arm or the entire chromosome such as occurs in some human leucocyte cell lines (Miles and O'Neill, 1969), or a break at the heterochromatin segment of the long arm with a fusion of the distal long arm to a small acrocentric as is seen in the cell line LK1D (Miles *et al.*, 1968).

The sequences of losses and gains for homologues in cancer cells appear to bear some relation to the heterochromatin (in the sense of late labelling chromatin) of the chromosomes. Late label per unit length of chromosomes is as follows: 4–5, 13–15, 6–12 + X, 3, 2, 16–18, 21–22, 1, 19–20 from largest to smallest amounts (Miles, 1970). This compares with 4–5, 1, 21–22 + Y, 13–15, 3, 2, 16–18, 19–20, 6–12 + X for losses and gains (21 cases with 5 or more cells). Groups or homologues 4–5, 13–15, 3, 2, 16–18, and 19–20 are in the same order. While chromosome No. 1 has a smaller total amount of late replicating chromatin, it has a very prominent late replicating segment of the long arm near the centromere.

It has been argued that heterochromatin segments are more apt to be broken (Gilbert *et al.*, 1962). This could lead to losses and to formation of marker chromosomes. Centric fusion may be preceded by a heterochromatin break in the short arms of the involved acrocentrics (White, 1954). Attenuated secondary constrictions have been noted in many cancer cells (Miles *et al.*, 1966; Miles, 1967*a*; Spiers and Baikie, 1968) and some of the more common of such constrictions (chromosomes Nos 1 and 16) are associated with heterochromatin segments. These constrictions may be of particular interest in view of their inducibility by viruses (O'Neill and Miles, 1969).

If, as has been postulated (O'Neill and Miles, 1970), pulverization results from forced mitotic condensation of chromosomes before DNA synthesis is complete, then late replicating segments would be more apt to be pulverized. The prominent heterochromatin segment on the long arm of chromosome No. 1 has been alluded to, and the long arm is particularly susceptible to pulverization (Miles and O'Neill, 1969). (This assumes, of course, that in some instances the appearance of pulverization may actually represent fragmentation of the affected chromosome.)

If indeed heterochromatin areas are preferentially affected, one possible explanation relates to the supposed genetic inertness of heterochromatin. It is possible that both euchromatin and heterochromatin areas are equally affected but that euchromatin damage tends more often to be lethal. Selection might then give the appearance of preferential damage to heterochromatin. One approach to answering this question might be to study the distribution of short-term damage effects, such as multiradials and fragments.

Conceivably the cytogenetic processes leading to karyotype abnormalities in cancer do not differ from those occurring in benign cells. Rather, these processes are simply greatly speeded up in many cancers.

It should be mentioned that some studies either do not confirm non-random changes or else report a somewhat different effect. Sandberg *et al.* (1968) did not find evidence for centric fusion in an analysis of 26 cases of human cancer.

Minkler, Gofman and Tandy (1970) report a marked excess of chromosome No. 16 in their series; other imbalances were not consistent. Their method of karyotyping is certainly more objective than that usually employed and it is possible that they have more often correctly assigned small submetacentric chromosomes to the 16 than to the 12 position. On the other hand, their series includes 17 cell lines, many of which may be of common origin (Gartler, 1968). There were only 11 cases with direct preparations. Perhaps, with more cases, other significant changes might have been detected. Moreover, Bender, Kastenbaum and Lever (1972) were not able to confirm the accuracy of the karyotyping method when employed on benign diploid cells.

I should like to express my appreciation for their help to Dr Frank J. O'Neill, Mr David Anderson and Mr Patrick Clapshaw.

This work was supported in part by Damon Runyon Memorial Fund for Cancer Research, Grant DRG798 and by National Cancer Institute Grant 5 RO1 CA 12668.

### REFERENCES

Atkin, N. B. & Baker, M. C. (1969) Possible Differences between the Karyotypes of Preinvasive Lesions and Malignant Tumours. *Br. J. Cancer*, **23**, 329.

Atkin, N. B. & Ross, A. J. (1960) Polyploidy in Human Tumours. *Nature, Lond.*, **187**, 579.

Bender, M. A., Kastenbaum, M. A. & Lever, C. S. (1972) Chromosome 16: A Specific Chromosomal Pathway for the Origin of Human Malignancy? *Br. J. Cancer*, **26**, 34

Gartler, S. M. (1968) Apparent HeLa Cell Contamination of Human Heteroploid Cell Lines. *Nature, Lond.*, **217**, 750.

Gilbert, C. W., Muldal, S., Lajtha, L. G. & Rowley, J. (1962) Time Sequence of Human Chromosome Duplication. *Nature, Lond.*, **195**, 869.

Ishihara, T., Kikuchi, Y. & Sandberg, A. A. (1963) Chromosomes of Twenty Cancer Effusions: Correlation of Karyotypic, Clinical and Pathologic Aspects. *J. natn. Cancer Inst.*, **30**, 1303.

LEVAN, A. (1966) Non-random Representation of Chromosome Types in Human Tumor Stemlines. *Hereditas*, **55**, 28.

LUBS, H. A. & CLARK, R. (1963) The Chromosome Complement of Human Solid Tumors. I. Gastrointestinal Tumors and Technic. *New Engl. J. Med.*, **268**, 907.

LUBS, H. A. & SALMON, J. H. (1965) The Chromosomal Complement of Human Solid Tumors. II. Karyotypes of Glial Tumors. *J. Neurosurg.*, **22**, 160.

MAKINO, S., SASAKI, M. S. & TONOMURA, A. (1964) Cytological Studies of Tumors. XL. Chromosome Studies in Fifty-two Human Tumors. *J. natn. Cancer Inst.*, **32**, 741.

MILES, C. P., GELLER, W. & O'NEILL, F. (1966) Chromosomes in Hodgkin's Disease and Other Malignant Lymphomas. *Cancer, N.Y.*, **19**, 1103.

MILES, C. P. (1967*a*) Chromosome Analysis of Solid Tumors. I. Twenty-eight Nonepithelial Tumors. *Cancer, N.Y.*, **20**, 1253.

MILES, C. P. (1967*b*) Chromosome Analysis of Solid Tumors, II. Twenty-six Epithelial Tumors. *Cancer, N.Y.*, **20**, 1274.

MILES, C. P. (1970) Labeling and Other Effects of Actinomycin D on Human Chromosomes. *Proc. natn. Acad. Sci. U.S.A.*, **65**, 585.

MILES, C. P. & O'NEILL, F. (1969) $^3$H Labeling Patterns of Permanent Cell Line Chromosomes showing Pulverization or Accentuated Secondary Constrictions. *J. cell Biol.*, **40**, 553.

MILES, C. P., O'NEILL, F., ARMSTRONG, D., CLARKSON, B. & KEANE, J. (1968) Chromosome Patterns of Human Leukocyte Established Cell Lines. *Cancer Res.*, **28**, 481.

MILES, C. P. & WOLINSKA, W. (1973) A Comparative Analysis of Chromosomes and Diagnostic Cytology in Effusions from 58 Cancer Patients. *Cancer, N.Y.*, **32**, 1458.

MINKLER, J. L., GOFMAN, J. W. & TANDY, R. K. (1970) A Specific Common Chromosomal Pathway for the Origin of Human Malignancy—II. *Br. J. Cancer*, **24**, 726.

MULDAL, S., ELEJALDE, R. & HARVEY, P. W. (1971) Specific Chromosome anomaly Associated with Autonomous and Cancerous Development in Man. *Nature, Lond.*, **229**, 48.

O'NEILL, F. J. & MILES, C. P. (1969) Chromosome Changes Induced by Herpes Simplex, Types 1 and 2 in Human Cells. *Nature, Lond.*, **223**, 851.

O'NEILL, F. J. & MILES, C. P. (1970) Virus-induced Chromosome Pulverization in Human Diploid Cells. *Proc. Soc. exp. Biol. Med.*, **134**, 825.

RICCI, N., PUNTURIERI, E., BOSI, L. & CASTOLDI, G. L. (1962) Chromosomes of Sternberg–Reed Cells. *Lancet*, ii, 564.

SANDBERG, A. A., BROSS, I. D. J., TAKAGI, N. & SCHMIDT, M. L. (1968) Chromosomes and Causation of Human Cancer and Leukemia IV. Vectorial Analysis. *Cancer, N.Y.*, **21**, 77.

SANDBERG, A. A. & HOSSFELD, D. K. (1970) Chromosomal Abnormalities in Human Neoplasia. *Ann. rev. Med.*, **21**, 379.

SPIERS, A. S. D. & BAIKIE, A. G. (1968) Cytogenetic Studies in the Malignant Lymphomas and Related Neoplasms. *Cancer, N.Y.*, **22**, 193.

SPRIGGS, A. I., BODDINGTON, M. M. & CLARKE, C. M. (1962) Carcinoma-in-situ of the Cervix Uteri, Some Cytogenetic Observations. *Lancet*, i, 1383.

VAN STEENIS, H. (1954) Chromosomes and Cancer. *Nature, Lond.*, **209**, 819.

WHITE, M. J. D. (1954) *Animal Cytology and Evolution*. 2nd Edn. Massachusetts: Cambridge University Press.